# Measurement of WZ and ZZ production in pp collisions at $\sqrt{s} = 8\,\text {TeV}$ in final states with b-tagged jets

**DOI:** 10.1140/epjc/s10052-014-2973-5

**Published:** 2014-08-07

**Authors:** The CMS Collaboration, S. Chatrchyan, V. Khachatryan, A. M. Sirunyan, A. Tumasyan, W. Adam, T. Bergauer, M. Dragicevic, J. Erö, C. Fabjan, M. Friedl, R. Frühwirth, V. M. Ghete, C. Hartl, N. Hörmann, J. Hrubec, M. Jeitler, W. Kiesenhofer, V. Knünz, M. Krammer, I. Krätschmer, D. Liko, I. Mikulec, D. Rabady, B. Rahbaran, H. Rohringer, R. Schöfbeck, J. Strauss, A. Taurok, W. Treberer-Treberspurg, W. Waltenberger, C. -E. Wulz, V. Mossolov, N. Shumeiko, J. Suarez Gonzalez, S. Alderweireldt, M. Bansal, S. Bansal, T. Cornelis, E. A. De Wolf, X. Janssen, A. Knutsson, S. Luyckx, S. Ochesanu, B. Roland, R. Rougny, H. Van Haevermaet, P. Van Mechelen, N. Van Remortel, A. Van Spilbeeck, F. Blekman, S. Blyweert, J. D’Hondt, N. Heracleous, A. Kalogeropoulos, J. Keaveney, T. J. Kim, S. Lowette, M. Maes, A. Olbrechts, D. Strom, S. Tavernier, W. Van Doninck, P. Van Mulders, G. P. Van Onsem, I. Villella, C. Caillol, B. Clerbaux, G. De Lentdecker, L. Favart, A. P. R. Gay, A. Léonard, P. E. Marage, A. Mohammadi, L. Perniè, T. Reis, T. Seva, L. Thomas, C. Vander Velde, P. Vanlaer, J. Wang, V. Adler, K. Beernaert, L. Benucci, A. Cimmino, S. Costantini, S. Crucy, S. Dildick, G. Garcia, B. Klein, J. Lellouch, J. Mccartin, A. A. Ocampo Rios, D. Ryckbosch, S. Salva Diblen, M. Sigamani, N. Strobbe, F. Thyssen, M. Tytgat, S. Walsh, E. Yazgan, N. Zaganidis, S. Basegmez, C. Beluffi, G. Bruno, R. Castello, A. Caudron, L. Ceard, G. G. Da Silveira, C. Delaere, T. du Pree, D. Favart, L. Forthomme, A. Giammanco, J. Hollar, P. Jez, M. Komm, V. Lemaitre, J. Liao, O. Militaru, C. Nuttens, D. Pagano, A. Pin, K. Piotrzkowski, A. Popov, L. Quertenmont, M. Selvaggi, M. Vidal Marono, J. M. Vizan Garcia, N. Beliy, T. Caebergs, E. Daubie, G. H. Hammad, G. A. Alves, M. Correa Martins Junior, T. Martins, M. E. Pol, M. H. G. Souza, W. L. Aldá Júnior, W. Carvalho, J. Chinellato, A. Custódio, E. M. Da Costa, D. De Jesus Damiao, C. De Oliveira Martins, S. Fonseca De Souza, H. Malbouisson, M. Malek, D. Matos Figueiredo, L. Mundim, H. Nogima, W. L. Prado Da Silva, J. Santaolalla, A. Santoro, A. Sznajder, E. J. Tonelli Manganote, A. Vilela Pereira, F. A. Dias, T. R. Fernandez Perez Tomei, S. F. Novaes, Sandra S. Padula, C. A. Bernardes, E. M. Gregores, P. G. Mercadante, V. Genchev, P. Iaydjiev, A. Marinov, S. Piperov, M. Rodozov, G. Sultanov, M. Vutova, A. Dimitrov, I. Glushkov, R. Hadjiiska, V. Kozhuharov, L. Litov, B. Pavlov, P. Petkov, J. G. Bian, G. M. Chen, H. S. Chen, M. Chen, R. Du, C. H. Jiang, D. Liang, S. Liang, X. Meng, R. Plestina, J. Tao, X. Wang, Z. Wang, C. Asawatangtrakuldee, Y. Ban, Y. Guo, Q. Li, W. Li, S. Liu, Y. Mao, S. J. Qian, D. Wang, L. Zhang, W. Zou, C. Avila, L. F. Chaparro Sierra, C. Florez, J. P. Gomez, B. Gomez Moreno, J. C. Sanabria, N. Godinovic, D. Lelas, D. Polic, I. Puljak, Z. Antunovic, M. Kovac, V. Brigljevic, K. Kadija, J. Luetic, D. Mekterovic, S. Morovic, L. Tikvica, A. Attikis, G. Mavromanolakis, J. Mousa, C. Nicolaou, F. Ptochos, P. A. Razis, M. Finger, M. Finger, Y. Assran, S. Elgammal, A. Ellithi Kamel, M. A. Mahmoud, A. Mahrous, A. Radi, M. Kadastik, M. Müntel, M. Murumaa, M. Raidal, A. Tiko, P. Eerola, G. Fedi, M. Voutilainen, J. Härkönen, V. Karimäki, R. Kinnunen, M. J. Kortelainen, T. Lampén, K. Lassila-Perini, S. Lehti, T. Lindén, P. Luukka, T. Mäenpää, T. Peltola, E. Tuominen, J. Tuominiemi, E. Tuovinen, L. Wendland, T. Tuuva, M. Besancon, F. Couderc, M. Dejardin, D. Denegri, B. Fabbro, J. L. Faure, F. Ferri, S. Ganjour, A. Givernaud, P. Gras, G. Hamel de Monchenault, P. Jarry, E. Locci, J. Malcles, A. Nayak, J. Rander, A. Rosowsky, M. Titov, S. Baffioni, F. Beaudette, P. Busson, C. Charlot, N. Daci, T. Dahms, M. Dalchenko, L. Dobrzynski, N. Filipovic, A. Florent, R. Granier de Cassagnac, L. Mastrolorenzo, P. Miné, C. Mironov, I. N. Naranjo, M. Nguyen, C. Ochando, P. Paganini, D. Sabes, R. Salerno, J. b. Sauvan, Y. Sirois, C. Veelken, Y. Yilmaz, A. Zabi, J. -L. Agram, J. Andrea, D. Bloch, J. -M. Brom, E. C. Chabert, C. Collard, E. Conte, F. Drouhin, J. -C. Fontaine, D. Gelé, U. Goerlach, C. Goetzmann, P. Juillot, A. -C. Le Bihan, P. Van Hove, S. Gadrat, S. Beauceron, N. Beaupere, G. Boudoul, S. Brochet, C. A. Carrillo Montoya, J. Chasserat, R. Chierici, D. Contardo, P. Depasse, H. El Mamouni, J. Fan, J. Fay, S. Gascon, M. Gouzevitch, B. Ille, T. Kurca, M. Lethuillier, L. Mirabito, S. Perries, J. D. Ruiz Alvarez, L. Sgandurra, V. Sordini, M. Vander Donckt, P. Verdier, S. Viret, H. Xiao, Z. Tsamalaidze, C. Autermann, S. Beranek, M. Bontenackels, B. Calpas, M. Edelhoff, L. Feld, O. Hindrichs, K. Klein, A. Ostapchuk, A. Perieanu, F. Raupach, J. Sammet, S. Schael, D. Sprenger, H. Weber, B. Wittmer, V. Zhukov, M. Ata, J. Caudron, E. Dietz-Laursonn, D. Duchardt, M. Erdmann, R. Fischer, A. Güth, T. Hebbeker, C. Heidemann, K. Hoepfner, D. Klingebiel, S. Knutzen, P. Kreuzer, M. Merschmeyer, A. Meyer, M. Olschewski, K. Padeken, P. Papacz, H. Reithler, S. A. Schmitz, L. Sonnenschein, D. Teyssier, S. Thüer, M. Weber, V. Cherepanov, Y. Erdogan, G. Flügge, H. Geenen, M. Geisler, W. Haj Ahmad, F. Hoehle, B. Kargoll, T. Kress, Y. Kuessel, J. Lingemann, A. Nowack, I. M. Nugent, L. Perchalla, O. Pooth, A. Stahl, I. Asin, N. Bartosik, J. Behr, W. Behrenhoff, U. Behrens, A. J. Bell, M. Bergholz, A. Bethani, K. Borras, A. Burgmeier, A. Cakir, L. Calligaris, A. Campbell, S. Choudhury, F. Costanza, C. Diez Pardos, S. Dooling, T. Dorland, G. Eckerlin, D. Eckstein, T. Eichhorn, G. Flucke, A. Geiser, A. Grebenyuk, P. Gunnellini, S. Habib, J. Hauk, G. Hellwig, M. Hempel, D. Horton, H. Jung, M. Kasemann, P. Katsas, J. Kieseler, C. Kleinwort, M. Krämer, D. Krücker, W. Lange, J. Leonard, K. Lipka, W. Lohmann, B. Lutz, R. Mankel, I. Marfin, I. -A. Melzer-Pellmann, A. B. Meyer, J. Mnich, A. Mussgiller, S. Naumann-Emme, O. Novgorodova, F. Nowak, E. Ntomari, H. Perrey, A. Petrukhin, D. Pitzl, R. Placakyte, A. Raspereza, P. M. Ribeiro Cipriano, C. Riedl, E. Ron, M. Ö. Sahin, J. Salfeld-Nebgen, P. Saxena, R. Schmidt, T. Schoerner-Sadenius, M. Schröder, M. Stein, A. D. R. Vargas Trevino, R. Walsh, C. Wissing, M. Aldaya Martin, V. Blobel, H. Enderle, J. Erfle, E. Garutti, K. Goebel, M. Görner, M. Gosselink, J. Haller, R. S. Höing, H. Kirschenmann, R. Klanner, R. Kogler, J. Lange, T. Lapsien, T. Lenz, I. Marchesini, J. Ott, T. Peiffer, N. Pietsch, D. Rathjens, C. Sander, H. Schettler, P. Schleper, E. Schlieckau, A. Schmidt, M. Seidel, J. Sibille, V. Sola, H. Stadie, G. Steinbrück, D. Troendle, E. Usai, L. Vanelderen, C. Barth, C. Baus, J. Berger, C. Böser, E. Butz, T. Chwalek, W. De Boer, A. Descroix, A. Dierlamm, M. Feindt, M. Guthoff, F. Hartmann, T. Hauth, H. Held, K. H. Hoffmann, U. Husemann, I. Katkov, A. Kornmayer, E. Kuznetsova, P. Lobelle Pardo, D. Martschei, M. U. Mozer, Th. Müller, M. Niegel, A. Nürnberg, O. Oberst, G. Quast, K. Rabbertz, F. Ratnikov, S. Röcker, F. -P. Schilling, G. Schott, H. J. Simonis, F. M. Stober, R. Ulrich, J. Wagner-Kuhr, S. Wayand, T. Weiler, R. Wolf, M. Zeise, G. Anagnostou, G. Daskalakis, T. Geralis, S. Kesisoglou, A. Kyriakis, D. Loukas, A. Markou, C. Markou, A. Psallidas, I. Topsis-Giotis, L. Gouskos, A. Panagiotou, N. Saoulidou, E. Stiliaris, X. Aslanoglou, I. Evangelou, G. Flouris, C. Foudas, J. Jones, P. Kokkas, N. Manthos, I. Papadopoulos, E. Paradas, G. Bencze, C. Hajdu, P. Hidas, D. Horvath, F. Sikler, V. Veszpremi, G. Vesztergombi, A. J. Zsigmond, N. Beni, S. Czellar, J. Molnar, J. Palinkas, Z. Szillasi, J. Karancsi, P. Raics, Z. L. Trocsanyi, B. Ujvari, S. K. Swain, S. B. Beri, V. Bhatnagar, N. Dhingra, R. Gupta, M. Kaur, M. Mittal, N. Nishu, A. Sharma, J. B. Singh, Ashok Kumar, Arun Kumar, S. Ahuja, A. Bhardwaj, B. C. Choudhary, A. Kumar, S. Malhotra, M. Naimuddin, K. Ranjan, V. Sharma, R. K. Shivpuri, S. Banerjee, S. Bhattacharya, K. Chatterjee, S. Dutta, B. Gomber, Sa. Jain, Sh. Jain, R. Khurana, A. Modak, S. Mukherjee, D. Roy, S. Sarkar, M. Sharan, A. P. Singh, A. Abdulsalam, D. Dutta, S. Kailas, V. Kumar, A. K. Mohanty, L. M. Pant, P. Shukla, A. Topkar, T. Aziz, R. M. Chatterjee, S. Ganguly, S. Ghosh, M. Guchait, A. Gurtu, G. Kole, S. Kumar, M. Maity, G. Majumder, K. Mazumdar, G. B. Mohanty, B. Parida, K. Sudhakar, N. Wickramage, S. Dugad, H. Arfaei, H. Bakhshiansohi, H. Behnamian, S. M. Etesami, A. Fahim, A. Jafari, M. Khakzad, M. Mohammadi Najafabadi, M. Naseri, S. Paktinat Mehdiabadi, B. Safarzadeh, M. Zeinali, M. Grunewald, M. Abbrescia, L. Barbone, C. Calabria, S. S. Chhibra, A. Colaleo, D. Creanza, N. De Filippis, M. De Palma, L. Fiore, G. Iaselli, G. Maggi, M. Maggi, B. Marangelli, S. My, S. Nuzzo, N. Pacifico, A. Pompili, G. Pugliese, R. Radogna, G. Selvaggi, L. Silvestris, G. Singh, R. Venditti, P. Verwilligen, G. Zito, G. Abbiendi, A. C. Benvenuti, D. Bonacorsi, S. Braibant-Giacomelli, L. Brigliadori, R. Campanini, P. Capiluppi, A. Castro, F. R. Cavallo, G. Codispoti, M. Cuffiani, G. M. Dallavalle, F. Fabbri, A. Fanfani, D. Fasanella, P. Giacomelli, C. Grandi, L. Guiducci, S. Marcellini, G. Masetti, M. Meneghelli, A. Montanari, F. L. Navarria, F. Odorici, A. Perrotta, F. Primavera, A. M. Rossi, T. Rovelli, G. P. Siroli, N. Tosi, R. Travaglini, S. Albergo, G. Cappello, M. Chiorboli, S. Costa, F. Giordano, R. Potenza, A. Tricomi, C. Tuve, G. Barbagli, V. Ciulli, C. Civinini, R. D’Alessandro, E. Focardi, E. Gallo, S. Gonzi, V. Gori, P. Lenzi, M. Meschini, S. Paoletti, G. Sguazzoni, A. Tropiano, L. Benussi, S. Bianco, D. Piccolo, P. Fabbricatore, R. Ferretti, F. Ferro, M. Lo Vetere, R. Musenich, E. Robutti, S. Tosi, M. E. Dinardo, S. Fiorendi, S. Gennai, R. Gerosa, A. Ghezzi, P. Govoni, M. T. Lucchini, S. Malvezzi, R. A. Manzoni, A. Martelli, B. Marzocchi, D. Menasce, L. Moroni, M. Paganoni, D. Pedrini, S. Ragazzi, N. Redaelli, T. Tabarelli de Fatis, S. Buontempo, N. Cavallo, S. Di Guida, F. Fabozzi, A. O. M. Iorio, L. Lista, S. Meola, M. Merola, P. Paolucci, P. Azzi, N. Bacchetta, D. Bisello, A. Branca, R. Carlin, P. Checchia, T. Dorigo, U. Dosselli, M. Galanti, F. Gasparini, U. Gasparini, P. Giubilato, A. Gozzelino, K. Kanishchev, S. Lacaprara, I. Lazzizzera, M. Margoni, A. T. Meneguzzo, J. Pazzini, M. Pegoraro, N. Pozzobon, P. Ronchese, F. Simonetto, E. Torassa, M. Tosi, A. Triossi, P. Zotto, A. Zucchetta, G. Zumerle, M. Gabusi, S. P. Ratti, C. Riccardi, P. Salvini, P. Vitulo, M. Biasini, G. M. Bilei, L. Fanò, P. Lariccia, G. Mantovani, M. Menichelli, F. Romeo, A. Saha, A. Santocchia, A. Spiezia, K. Androsov, P. Azzurri, G. Bagliesi, J. Bernardini, T. Boccali, G. Broccolo, R. Castaldi, M. A. Ciocci, R. Dell’Orso, S. Donato, F. Fiori, L. Foà, A. Giassi, M. T. Grippo, A. Kraan, F. Ligabue, T. Lomtadze, L. Martini, A. Messineo, C. S. Moon, F. Palla, A. Rizzi, A. Savoy-Navarro, A. T. Serban, P. Spagnolo, P. Squillacioti, R. Tenchini, G. Tonelli, A. Venturi, P. G. Verdini, C. Vernieri, L. Barone, F. Cavallari, D. Del Re, M. Diemoz, M. Grassi, C. Jorda, E. Longo, F. Margaroli, P. Meridiani, F. Micheli, S. Nourbakhsh, G. Organtini, R. Paramatti, S. Rahatlou, C. Rovelli, L. Soffi, P. Traczyk, N. Amapane, R. Arcidiacono, S. Argiro, M. Arneodo, R. Bellan, C. Biino, N. Cartiglia, S. Casasso, M. Costa, A. Degano, N. Demaria, C. Mariotti, S. Maselli, E. Migliore, V. Monaco, M. Musich, M. M. Obertino, G. Ortona, L. Pacher, N. Pastrone, M. Pelliccioni, A. Potenza, A. Romero, M. Ruspa, R. Sacchi, A. Solano, A. Staiano, U. Tamponi, S. Belforte, V. Candelise, M. Casarsa, F. Cossutti, G. Della Ricca, B. Gobbo, C. La Licata, M. Marone, D. Montanino, A. Penzo, A. Schizzi, T. Umer, A. Zanetti, S. Chang, T. Y. Kim, S. K. Nam, D. H. Kim, G. N. Kim, J. E. Kim, M. S. Kim, D. J. Kong, S. Lee, Y. D. Oh, H. Park, A. Sakharov, D. C. Son, J. Y. Kim, Zero J. Kim, S. Song, S. Choi, D. Gyun, B. Hong, M. Jo, H. Kim, Y. Kim, B. Lee, K. S. Lee, S. K. Park, Y. Roh, M. Choi, J. H. Kim, C. Park, I. C. Park, S. Park, G. Ryu, Y. Choi, Y. K. Choi, J. Goh, E. Kwon, J. Lee, H. Seo, I. Yu, A. Juodagalvis, J. R. Komaragiri, H. Castilla-Valdez, E. De La Cruz-Burelo, I. Heredia-de La Cruz, R. Lopez-Fernandez, J. Martínez-Ortega, A. Sanchez-Hernandez, L. M. Villasenor-Cendejas, S. Carrillo Moreno, F. Vazquez Valencia, H. A. Salazar Ibarguen, E. Casimiro Linares, A. Morelos Pineda, D. Krofcheck, P. H. Butler, R. Doesburg, S. Reucroft, A. Ahmad, M. Ahmad, M. I. Asghar, J. Butt, Q. Hassan, H. R. Hoorani, W. A. Khan, T. Khurshid, S. Qazi, M. A. Shah, M. Shoaib, H. Bialkowska, M. Bluj, B. Boimska, T. Frueboes, M. Górski, M. Kazana, K. Nawrocki, K. Romanowska-Rybinska, M. Szleper, G. Wrochna, P. Zalewski, G. Brona, K. Bunkowski, M. Cwiok, W. Dominik, K. Doroba, A. Kalinowski, M. Konecki, J. Krolikowski, M. Misiura, W. Wolszczak, P. Bargassa, C. Beirão Da Cruz E Silva, P. Faccioli, P. G. Ferreira Parracho, M. Gallinaro, F. Nguyen, J. Rodrigues Antunes, J. Seixas, J. Varela, P. Vischia, P. Bunin, I. Golutvin, I. Gorbunov, A. Kamenev, V. Karjavin, V. Konoplyanikov, V. Korenkov, A. Lanev, A. Malakhov, V. Matveev, P. Moisenz, V. Palichik, V. Perelygin, S. Shmatov, N. Skatchkov, V. Smirnov, E. Tikhonenko, A. Zarubin, V. Golovtsov, Y. Ivanov, V. Kim, P. Levchenko, V. Murzin, V. Oreshkin, I. Smirnov, V. Sulimov, L. Uvarov, S. Vavilov, A. Vorobyev, An. Vorobyev, Yu. Andreev, A. Dermenev, S. Gninenko, N. Golubev, M. Kirsanov, N. Krasnikov, A. Pashenkov, D. Tlisov, A. Toropin, V. Epshteyn, V. Gavrilov, N. Lychkovskaya, V. Popov, G. Safronov, S. Semenov, A. Spiridonov, V. Stolin, E. Vlasov, A. Zhokin, V. Andreev, M. Azarkin, I. Dremin, M. Kirakosyan, A. Leonidov, G. Mesyats, S. V. Rusakov, A. Vinogradov, A. Belyaev, E. Boos, M. Dubinin, L. Dudko, A. Ershov, A. Gribushin, V. Klyukhin, O. Kodolova, I. Lokhtin, S. Obraztsov, S. Petrushanko, V. Savrin, A. Snigirev, I. Azhgirey, I. Bayshev, S. Bitioukov, V. Kachanov, A. Kalinin, D. Konstantinov, V. Krychkine, V. Petrov, R. Ryutin, A. Sobol, L. Tourtchanovitch, S. Troshin, N. Tyurin, A. Uzunian, A. Volkov, P. Adzic, M. Djordjevic, M. Ekmedzic, J. Milosevic, M. Aguilar-Benitez, J. Alcaraz Maestre, C. Battilana, E. Calvo, M. Cerrada, M. Chamizo Llatas, N. Colino, B. De La Cruz, A. Delgado Peris, D. Domínguez Vázquez, C. Fernandez Bedoya, J. P. Fernández Ramos, A. Ferrando, J. Flix, M. C. Fouz, P. Garcia-Abia, O. Gonzalez Lopez, S. Goy Lopez, J. M. Hernandez, M. I. Josa, G. Merino, E. Navarro De Martino, A. Pérez-Calero Yzquierdo, J. Puerta Pelayo, A. Quintario Olmeda, I. Redondo, L. Romero, M. S. Soares, C. Willmott, C. Albajar, J. F. de Trocóniz, M. Missiroli, H. Brun, J. Cuevas, J. Fernandez Menendez, S. Folgueras, I. Gonzalez Caballero, L. Lloret Iglesias, J. A. Brochero Cifuentes, I. J. Cabrillo, A. Calderon, J. Duarte Campderros, M. Fernandez, G. Gomez, J. Gonzalez Sanchez, A. Graziano, A. Lopez Virto, J. Marco, R. Marco, C. Martinez Rivero, F. Matorras, F. J. Munoz Sanchez, J. Piedra Gomez, T. Rodrigo, A. Y. Rodríguez-Marrero, A. Ruiz-Jimeno, L. Scodellaro, I. Vila, R. Vilar Cortabitarte, D. Abbaneo, E. Auffray, G. Auzinger, M. Bachtis, P. Baillon, A. H. Ball, D. Barney, A. Benaglia, J. Bendavid, L. Benhabib, J. F. Benitez, C. Bernet, G. Bianchi, P. Bloch, A. Bocci, A. Bonato, O. Bondu, C. Botta, H. Breuker, T. Camporesi, G. Cerminara, T. Christiansen, J. A. Coarasa Perez, S. Colafranceschi, M. D’Alfonso, D. d’Enterria, A. Dabrowski, A. David, F. De Guio, A. De Roeck, S. De Visscher, M. Dobson, N. Dupont-Sagorin, A. Elliott-Peisert, J. Eugster, G. Franzoni, W. Funk, M. Giffels, D. Gigi, K. Gill, D. Giordano, M. Girone, M. Giunta, F. Glege, R. Gomez-Reino Garrido, S. Gowdy, R. Guida, J. Hammer, M. Hansen, P. Harris, J. Hegeman, V. Innocente, P. Janot, E. Karavakis, K. Kousouris, K. Krajczar, P. Lecoq, C. Lourenço, N. Magini, L. Malgeri, M. Mannelli, L. Masetti, F. Meijers, S. Mersi, E. Meschi, F. Moortgat, M. Mulders, P. Musella, L. Orsini, E. Palencia Cortezon, L. Pape, E. Perez, L. Perrozzi, A. Petrilli, G. Petrucciani, A. Pfeiffer, M. Pierini, M. Pimiä, D. Piparo, M. Plagge, A. Racz, W. Reece, G. Rolandi, M. Rovere, H. Sakulin, F. Santanastasio, C. Schäfer, C. Schwick, S. Sekmen, P. Siegrist, P. Silva, M. Simon, P. Sphicas, D. Spiga, J. Steggemann, B. Stieger, M. Stoye, D. Treille, A. Tsirou, G. I. Veres, J. R. Vlimant, H. K. Wöhri, W. D. Zeuner, W. Bertl, K. Deiters, W. Erdmann, R. Horisberger, Q. Ingram, H. C. Kaestli, S. König, D. Kotlinski, U. Langenegger, D. Renker, T. Rohe, F. Bachmair, L. Bäni, L. Bianchini, P. Bortignon, M. A. Buchmann, B. Casal, N. Chanon, A. Deisher, G. Dissertori, M. Dittmar, M. Donegà, M. Dünser, P. Eller, C. Grab, D. Hits, W. Lustermann, B. Mangano, A. C. Marini, P. Martinez Ruiz del Arbol, D. Meister, N. Mohr, C. Nägeli, P. Nef, F. Nessi-Tedaldi, F. Pandolfi, F. Pauss, M. Peruzzi, M. Quittnat, L. Rebane, F. J. Ronga, M. Rossini, A. Starodumov, M. Takahashi, K. Theofilatos, R. Wallny, H. A. Weber, C. Amsler, M. F. Canelli, V. Chiochia, A. De Cosa, C. Favaro, A. Hinzmann, T. Hreus, M. Ivova Rikova, B. Kilminster, B. Millan Mejias, J. Ngadiuba, P. Robmann, H. Snoek, S. Taroni, M. Verzetti, Y. Yang, M. Cardaci, K. H. Chen, C. Ferro, C. M. Kuo, S. W. Li, W. Lin, Y. J. Lu, R. Volpe, S. S. Yu, P. Bartalini, P. Chang, Y. H. Chang, Y. W. Chang, Y. Chao, K. F. Chen, P. H. Chen, C. Dietz, U. Grundler, W. -S. Hou, Y. Hsiung, K. Y. Kao, Y. J. Lei, Y. F. Liu, R. -S. Lu, D. Majumder, E. Petrakou, X. Shi, J. G. Shiu, Y. M. Tzeng, M. Wang, R. Wilken, B. Asavapibhop, N. Suwonjandee, A. Adiguzel, M. N. Bakirci, S. Cerci, C. Dozen, I. Dumanoglu, E. Eskut, S. Girgis, G. Gokbulut, E. Gurpinar, I. Hos, E. E. Kangal, A. Kayis Topaksu, G. Onengut, K. Ozdemir, S. Ozturk, A. Polatoz, K. Sogut, D. Sunar Cerci, B. Tali, H. Topakli, M. Vergili, I. V. Akin, T. Aliev, B. Bilin, S. Bilmis, M. Deniz, H. Gamsizkan, A. M. Guler, G. Karapinar, K. Ocalan, A. Ozpineci, M. Serin, R. Sever, U. E. Surat, M. Yalvac, M. Zeyrek, E. Gülmez, B. Isildak, M. Kaya, O. Kaya, S. Ozkorucuklu, H. Bahtiyar, E. Barlas, K. Cankocak, Y. O. Günaydin, F. I. Vardarlı, M. Yücel, L. Levchuk, P. Sorokin, J. J. Brooke, E. Clement, D. Cussans, H. Flacher, R. Frazier, J. Goldstein, M. Grimes, G. P. Heath, H. F. Heath, J. Jacob, L. Kreczko, C. Lucas, Z. Meng, D. M. Newbold, S. Paramesvaran, A. Poll, S. Senkin, V. J. Smith, T. Williams, K. W. Bell, C. Brew, R. M. Brown, D. J. A. Cockerill, J. A. Coughlan, K. Harder, S. Harper, J. Ilic, E. Olaiya, D. Petyt, C. H. Shepherd-Themistocleous, A. Thea, I. R. Tomalin, W. J. Womersley, S. D. Worm, M. Baber, R. Bainbridge, O. Buchmuller, D. Burton, D. Colling, N. Cripps, M. Cutajar, P. Dauncey, G. Davies, M. Della Negra, W. Ferguson, J. Fulcher, D. Futyan, A. Gilbert, A. Guneratne Bryer, G. Hall, Z. Hatherell, J. Hays, G. Iles, M. Jarvis, G. Karapostoli, M. Kenzie, R. Lane, R. Lucas, L. Lyons, A. -M. Magnan, J. Marrouche, B. Mathias, R. Nandi, J. Nash, A. Nikitenko, J. Pela, M. Pesaresi, K. Petridis, M. Pioppi, D. M. Raymond, S. Rogerson, A. Rose, C. Seez, P. Sharp, A. Sparrow, A. Tapper, M. Vazquez Acosta, T. Virdee, S. Wakefield, N. Wardle, J. E. Cole, P. R. Hobson, A. Khan, P. Kyberd, D. Leggat, D. Leslie, W. Martin, I. D. Reid, P. Symonds, L. Teodorescu, M. Turner, J. Dittmann, K. Hatakeyama, A. Kasmi, H. Liu, T. Scarborough, O. Charaf, S. I. Cooper, C. Henderson, P. Rumerio, A. Avetisyan, T. Bose, C. Fantasia, A. Heister, P. Lawson, D. Lazic, C. Richardson, J. Rohlf, D. Sperka, J. St. John, L. Sulak, J. Alimena, G. Christopher, D. Cutts, Z. Demiragli, A. Ferapontov, A. Garabedian, U. Heintz, S. Jabeen, G. Kukartsev, E. Laird, G. Landsberg, M. Luk, M. Narain, M. Segala, T. Sinthuprasith, T. Speer, J. Swanson, R. Breedon, G. Breto, M. Calderon De La Barca Sanchez, S. Chauhan, M. Chertok, J. Conway, R. Conway, P. T. Cox, R. Erbacher, M. Gardner, W. Ko, A. Kopecky, R. Lander, T. Miceli, M. Mulhearn, D. Pellett, J. Pilot, F. Ricci-Tam, B. Rutherford, M. Searle, S. Shalhout, J. Smith, M. Squires, M. Tripathi, S. Wilbur, R. Yohay, D. Cline, R. Cousins, S. Erhan, P. Everaerts, C. Farrell, M. Felcini, J. Hauser, M. Ignatenko, C. Jarvis, G. Rakness, P. Schlein, E. Takasugi, V. Valuev, M. Weber, J. Babb, R. Clare, J. Ellison, J. W. Gary, G. Hanson, J. Heilman, P. Jandir, F. Lacroix, O. R. Long, A. Luthra, M. Malberti, H. Nguyen, A. Shrinivas, J. Sturdy, S. Sumowidagdo, S. Wimpenny, W. Andrews, J. G. Branson, G. B. Cerati, S. Cittolin, R. T. D’Agnolo, D. Evans, A. Holzner, R. Kelley, D. Kovalskyi, M. Lebourgeois, J. Letts, I. Macneill, S. Padhi, C. Palmer, M. Pieri, M. Sani, V. Sharma, S. Simon, E. Sudano, M. Tadel, Y. Tu, A. Vartak, S. Wasserbaech, F. Würthwein, A. Yagil, J. Yoo, D. Barge, J. Bradmiller-Feld, C. Campagnari, T. Danielson, A. Dishaw, K. Flowers, M. Franco Sevilla, P. Geffert, C. George, F. Golf, J. Incandela, C. Justus, R. Magaña Villalba, N. Mccoll, V. Pavlunin, J. Richman, R. Rossin, D. Stuart, W. To, C. West, A. Apresyan, A. Bornheim, J. Bunn, Y. Chen, E. Di Marco, J. Duarte, D. Kcira, A. Mott, H. B. Newman, C. Pena, C. Rogan, M. Spiropulu, V. Timciuc, R. Wilkinson, S. Xie, R. Y. Zhu, V. Azzolini, A. Calamba, R. Carroll, T. Ferguson, Y. Iiyama, D. W. Jang, M. Paulini, J. Russ, H. Vogel, I. Vorobiev, J. P. Cumalat, B. R. Drell, W. T. Ford, A. Gaz, E. Luiggi Lopez, U. Nauenberg, J. G. Smith, K. Stenson, K. A. Ulmer, S. R. Wagner, J. Alexander, A. Chatterjee, J. Chu, N. Eggert, L. K. Gibbons, W. Hopkins, A. Khukhunaishvili, B. Kreis, N. Mirman, G. Nicolas Kaufman, J. R. Patterson, A. Ryd, E. Salvati, W. Sun, W. D. Teo, J. Thom, J. Thompson, J. Tucker, Y. Weng, L. Winstrom, P. Wittich, D. Winn, S. Abdullin, M. Albrow, J. Anderson, G. Apollinari, L. A. T. Bauerdick, A. Beretvas, J. Berryhill, P. C. Bhat, K. Burkett, J. N. Butler, V. Chetluru, H. W. K. Cheung, F. Chlebana, S. Cihangir, V. D. Elvira, I. Fisk, J. Freeman, Y. Gao, E. Gottschalk, L. Gray, D. Green, S. Grünendahl, O. Gutsche, D. Hare, R. M. Harris, J. Hirschauer, B. Hooberman, S. Jindariani, M. Johnson, U. Joshi, K. Kaadze, B. Klima, S. Kwan, J. Linacre, D. Lincoln, R. Lipton, T. Liu, J. Lykken, K. Maeshima, J. M. Marraffino, V. I. Martinez Outschoorn, S. Maruyama, D. Mason, P. McBride, K. Mishra, S. Mrenna, Y. Musienko, S. Nahn, C. Newman-Holmes, V. O’Dell, O. Prokofyev, N. Ratnikova, E. Sexton-Kennedy, S. Sharma, A. Soha, W. J. Spalding, L. Spiegel, L. Taylor, S. Tkaczyk, N. V. Tran, L. Uplegger, E. W. Vaandering, R. Vidal, A. Whitbeck, J. Whitmore, W. Wu, F. Yang, J. C. Yun, D. Acosta, P. Avery, D. Bourilkov, T. Cheng, S. Das, M. De Gruttola, G. P. Di Giovanni, D. Dobur, R. D. Field, M. Fisher, Y. Fu, I. K. Furic, J. Hugon, B. Kim, J. Konigsberg, A. Korytov, A. Kropivnitskaya, T. Kypreos, J. F. Low, K. Matchev, P. Milenovic, G. Mitselmakher, L. Muniz, A. Rinkevicius, L. Shchutska, N. Skhirtladze, M. Snowball, J. Yelton, M. Zakaria, V. Gaultney, S. Hewamanage, S. Linn, P. Markowitz, G. Martinez, J. L. Rodriguez, T. Adams, A. Askew, J. Bochenek, J. Chen, B. Diamond, J. Haas, S. Hagopian, V. Hagopian, K. F. Johnson, H. Prosper, V. Veeraraghavan, M. Weinberg, M. M. Baarmand, B. Dorney, M. Hohlmann, H. Kalakhety, F. Yumiceva, M. R. Adams, L. Apanasevich, V. E. Bazterra, R. R. Betts, I. Bucinskaite, R. Cavanaugh, O. Evdokimov, L. Gauthier, C. E. Gerber, D. J. Hofman, S. Khalatyan, P. Kurt, D. H. Moon, C. O’Brien, C. Silkworth, P. Turner, N. Varelas, U. Akgun, E. A. Albayrak, B. Bilki, W. Clarida, K. Dilsiz, F. Duru, M. Haytmyradov, J. -P. Merlo, H. Mermerkaya, A. Mestvirishvili, A. Moeller, J. Nachtman, H. Ogul, Y. Onel, F. Ozok, R. Rahmat, S. Sen, P. Tan, E. Tiras, J. Wetzel, T. Yetkin, K. Yi, B. A. Barnett, B. Blumenfeld, S. Bolognesi, D. Fehling, A. V. Gritsan, P. Maksimovic, C. Martin, M. Swartz, P. Baringer, A. Bean, G. Benelli, J. Gray, R. P. Kenny, M. Murray, D. Noonan, S. Sanders, J. Sekaric, R. Stringer, Q. Wang, J. S. Wood, A. F. Barfuss, I. Chakaberia, A. Ivanov, S. Khalil, M. Makouski, Y. Maravin, L. K. Saini, S. Shrestha, I. Svintradze, J. Gronberg, D. Lange, F. Rebassoo, D. Wright, A. Baden, B. Calvert, S. C. Eno, J. A. Gomez, N. J. Hadley, R. G. Kellogg, T. Kolberg, Y. Lu, M. Marionneau, A. C. Mignerey, K. Pedro, A. Skuja, J. Temple, M. B. Tonjes, S. C. Tonwar, A. Apyan, R. Barbieri, G. Bauer, W. Busza, I. A. Cali, M. Chan, L. Di Matteo, V. Dutta, G. Gomez Ceballos, M. Goncharov, D. Gulhan, M. Klute, Y. S. Lai, Y. -J. Lee, A. Levin, P. D. Luckey, T. Ma, C. Paus, D. Ralph, C. Roland, G. Roland, G. S. F. Stephans, F. Stöckli, K. Sumorok, D. Velicanu, J. Veverka, B. Wyslouch, M. Yang, A. S. Yoon, M. Zanetti, V. Zhukova, B. Dahmes, A. De Benedetti, A. Gude, S. C. Kao, K. Klapoetke, Y. Kubota, J. Mans, N. Pastika, R. Rusack, A. Singovsky, N. Tambe, J. Turkewitz, J. G. Acosta, L. M. Cremaldi, R. Kroeger, S. Oliveros, L. Perera, D. A. Sanders, D. Summers, E. Avdeeva, K. Bloom, S. Bose, D. R. Claes, A. Dominguez, R. Gonzalez Suarez, J. Keller, D. Knowlton, I. Kravchenko, J. Lazo-Flores, S. Malik, F. Meier, G. R. Snow, J. Dolen, A. Godshalk, I. Iashvili, S. Jain, A. Kharchilava, A. Kumar, S. Rappoccio, G. Alverson, E. Barberis, D. Baumgartel, M. Chasco, J. Haley, A. Massironi, D. Nash, T. Orimoto, D. Trocino, D. Wood, J. Zhang, A. Anastassov, K. A. Hahn, A. Kubik, L. Lusito, N. Mucia, N. Odell, B. Pollack, A. Pozdnyakov, M. Schmitt, S. Stoynev, K. Sung, M. Velasco, S. Won, D. Berry, A. Brinkerhoff, K. M. Chan, A. Drozdetskiy, M. Hildreth, C. Jessop, D. J. Karmgard, N. Kellams, J. Kolb, K. Lannon, W. Luo, S. Lynch, N. Marinelli, D. M. Morse, T. Pearson, M. Planer, R. Ruchti, J. Slaunwhite, N. Valls, M. Wayne, M. Wolf, A. Woodard, L. Antonelli, B. Bylsma, L. S. Durkin, S. Flowers, C. Hill, R. Hughes, K. Kotov, T. Y. Ling, D. Puigh, M. Rodenburg, G. Smith, C. Vuosalo, B. L. Winer, H. Wolfe, H. W. Wulsin, E. Berry, P. Elmer, V. Halyo, P. Hebda, A. Hunt, P. Jindal, S. A. Koay, P. Lujan, D. Marlow, T. Medvedeva, M. Mooney, J. Olsen, P. Piroué, X. Quan, A. Raval, H. Saka, D. Stickland, C. Tully, J. S. Werner, S. C. Zenz, A. Zuranski, E. Brownson, A. Lopez, H. Mendez, J. E. Ramirez Vargas, E. Alagoz, D. Benedetti, G. Bolla, D. Bortoletto, M. De Mattia, A. Everett, Z. Hu, M. K. Jha, M. Jones, K. Jung, M. Kress, N. Leonardo, D. Lopes Pegna, V. Maroussov, P. Merkel, D. H. Miller, N. Neumeister, B. C. Radburn-Smith, I. Shipsey, D. Silvers, A. Svyatkovskiy, F. Wang, W. Xie, L. Xu, H. D. Yoo, J. Zablocki, Y. Zheng, N. Parashar, A. Adair, B. Akgun, K. M. Ecklund, F. J. M. Geurts, W. Li, B. Michlin, B. P. Padley, R. Redjimi, J. Roberts, J. Zabel, B. Betchart, A. Bodek, R. Covarelli, P. de Barbaro, R. Demina, Y. Eshaq, T. Ferbel, A. Garcia-Bellido, P. Goldenzweig, J. Han, A. Harel, D. C. Miner, G. Petrillo, D. Vishnevskiy, M. Zielinski, A. Bhatti, R. Ciesielski, L. Demortier, K. Goulianos, G. Lungu, C. Mesropian, S. Arora, A. Barker, J. P. Chou, C. Contreras-Campana, E. Contreras-Campana, D. Duggan, D. Ferencek, Y. Gershtein, R. Gray, E. Halkiadakis, D. Hidas, A. Lath, S. Panwalkar, M. Park, R. Patel, V. Rekovic, J. Robles, S. Salur, S. Schnetzer, C. Seitz, S. Somalwar, R. Stone, S. Thomas, P. Thomassen, M. Walker, K. Rose, S. Spanier, Z. C. Yang, A. York, O. Bouhali, R. Eusebi, W. Flanagan, J. Gilmore, T. Kamon, V. Khotilovich, V. Krutelyov, R. Montalvo, I. Osipenkov, Y. Pakhotin, A. Perloff, J. Roe, A. Safonov, T. Sakuma, I. Suarez, A. Tatarinov, D. Toback, N. Akchurin, C. Cowden, J. Damgov, C. Dragoiu, P. R. Dudero, J. Faulkner, K. Kovitanggoon, S. Kunori, S. W. Lee, T. Libeiro, I. Volobouev, E. Appelt, A. G. Delannoy, S. Greene, A. Gurrola, W. Johns, C. Maguire, A. Melo, M. Sharma, P. Sheldon, B. Snook, S. Tuo, J. Velkovska, M. W. Arenton, S. Boutle, B. Cox, B. Francis, J. Goodell, R. Hirosky, A. Ledovskoy, H. Li, C. Lin, C. Neu, J. Wood, S. Gollapinni, R. Harr, P. E. Karchin, C. Kottachchi Kankanamge Don, P. Lamichhane, D. A. Belknap, L. Borrello, D. Carlsmith, M. Cepeda, S. Dasu, S. Duric, E. Friis, M. Grothe, R. Hall-Wilton, M. Herndon, A. Hervé, P. Klabbers, J. Klukas, A. Lanaro, C. Lazaridis, A. Levine, R. Loveless, A. Mohapatra, I. Ojalvo, T. Perry, G. A. Pierro, G. Polese, I. Ross, T. Sarangi, A. Savin, W. H. Smith, N. Woods

**Affiliations:** 1 Yerevan Physics Institute, Yerevan, Armenia; 2 Institut für Hochenergiephysik der OeAW, Wien, Austria; 3 National Centre for Particle and High Energy Physics, Minsk, Belarus; 4 Universiteit Antwerpen, Antwerpen, Belgium; 5 Vrije Universiteit Brussel, Brussel, Belgium; 6 Université Libre de Bruxelles, Bruxelles, Belgium; 7 Ghent University, Ghent, Belgium; 8 Université Catholique de Louvain, Louvain-la-Neuve, Belgium; 9 Université de Mons, Mons, Belgium; 10 Centro Brasileiro de Pesquisas Fisicas, Rio de Janeiro, Brazil; 11 Universidade do Estado do Rio de Janeiro, Rio de Janeiro, Brazil; 12 Universidade Estadual Paulista, São Paulo, Brazil; 13 Universidade Federal do ABC, São Paulo, Brazil; 14 Institute for Nuclear Research and Nuclear Energy, Sofia, Bulgaria; 15 University of Sofia, Sofia, Bulgaria; 16 Institute of High Energy Physics, Beijing, China; 17 State Key Laboratory of Nuclear Physics and Technology, Peking University, Beijing, China; 18 Universidad de Los Andes, Bogota, Colombia; 19 Technical University of Split, Split, Croatia; 20 University of Split, Split, Croatia; 21 Institute Rudjer Boskovic, Zagreb, Croatia; 22 University of Cyprus, Nicosia, Cyprus; 23 Charles University, Prague, Czech Republic; 24 Academy of Scientific Research and Technology of the Arab Republic of Egypt, Egyptian Network of High Energy Physics, Cairo, Egypt; 25 National Institute of Chemical Physics and Biophysics, Tallinn, Estonia; 26 Department of Physics, University of Helsinki, Helsinki, Finland; 27 Helsinki Institute of Physics, Helsinki, Finland; 28 Lappeenranta University of Technology, Lappeenranta, Finland; 29 DSM/IRFU, CEA/Saclay, Gif-sur-Yvette, France; 30 Laboratoire Leprince-Ringuet, Ecole Polytechnique, IN2P3-CNRS Palaiseau, France; 31 Institut Pluridisciplinaire Hubert Curien, Université de Strasbourg, Université de Haute Alsace Mulhouse, CNRS/IN2P3, Strasbourg, France; 32 Centre de Calcul de l’Institut National de Physique Nucleaire et de Physique des Particules, CNRS/IN2P3 Villeurbanne, France; 33 Institut de Physique Nucléaire de Lyon, Université de Lyon, Université Claude Bernard Lyon 1, CNRS-IN2P3, Villeurbanne, France; 34 Institute of High Energy Physics and Informatization, Tbilisi State University, Tbilisi, Georgia; 35 RWTH Aachen University, I. Physikalisches Institut, Aachen, Germany; 36 RWTH Aachen University, III. Physikalisches Institut A, Aachen, Germany; 37 RWTH Aachen University, III. Physikalisches Institut B, Aachen, Germany; 38 Deutsches Elektronen-Synchrotron, Hamburg, Germany; 39 University of Hamburg, Hamburg, Germany; 40 Institut für Experimentelle Kernphysik, Karlsruhe, Germany; 41 Institute of Nuclear and Particle Physics (INPP), NCSR Demokritos, Aghia Paraskevi, Greece; 42 University of Athens, Athens, Greece; 43 University of Ioánnina, Ioánnina, Greece; 44 Wigner Research Centre for Physics, Budapest, Hungary; 45 Institute of Nuclear Research ATOMKI, Debrecen, Hungary; 46 University of Debrecen, Debrecen, Hungary; 47 National Institute of Science Education and Research, Bhubaneswar, India; 48 Panjab University, Chandigarh, India; 49 University of Delhi, Delhi, India; 50 Saha Institute of Nuclear Physics, Kolkata, India; 51 Bhabha Atomic Research Centre, Mumbai, India; 52 Tata Institute of Fundamental Research - EHEP, Mumbai, India; 53 Tata Institute of Fundamental Research - HECR, Mumbai, India; 54 Institute for Research in Fundamental Sciences (IPM), Tehran, Iran; 55 University College Dublin, Dublin, Ireland; 56 INFN Sezione di Bari, Bari, Italy; 57 Università di Bari, Bari, Italy; 58 Politecnico di Bari, Bari, Italy; 59 INFN Sezione di Bologna, Bologna, Italy; 60 Università di Bologna, Bologna, Italy; 61 INFN Sezione di Catania, Catania, Italy; 62 Università di Catania, Catania, Italy; 63 INFN Sezione di Firenze, Firenze, Italy; 64 Università di Firenze, Firenze, Italy; 65 INFN Laboratori Nazionali di Frascati, Frascati, Italy; 66 INFN Sezione di Genova, Genova, Italy; 67 Università di Genova, Genova, Italy; 68 INFN Sezione di Milano-Bicocca, Milano, Italy; 69 Università di Milano-Bicocca, Milano, Italy; 70 INFN Sezione di Napoli, Napoli, Italy; 71 Università di Napoli ’Federico II’, Napoli, Italy; 72 Università della Basilicata (Potenza), Napoli, Italy; 73 Università G. Marconi (Roma), Napoli, Italy; 74 INFN Sezione di Padova, Padova, Italy; 75 Università di Padova, Padova, Italy; 76 Università di Trento (Trento), Padova, Italy; 77 INFN Sezione di Pavia, Pavia, Italy; 78 Università di Pavia, Pavia, Italy; 79 INFN Sezione di Perugia, Perugia, Italy; 80 Università di Perugia, Perugia, Italy; 81 INFN Sezione di Pisa, Pisa, Italy; 82 Università di Pisa, Pisa, Italy; 83 Scuola Normale Superiore di Pisa, Pisa, Italy; 84 INFN Sezione di Roma, Roma, Italy; 85 Università di Roma, Roma, Italy; 86 INFN Sezione di Torino, Torino, Italy; 87 Università di Torino, Torino, Italy; 88 Università del Piemonte Orientale (Novara), Torino, Italy; 89 INFN Sezione di Trieste, Trieste, Italy; 90 Università di Trieste, Trieste, Italy; 91 Kangwon National University, Chunchon, Korea; 92 Kyungpook National University, Daegu, Korea; 93 Chonnam National University, Institute for Universe and Elementary Particles, Kwangju, Korea; 94 Korea University, Seoul, Korea; 95 University of Seoul, Seoul, Korea; 96 Sungkyunkwan University, Suwon, Korea; 97 Vilnius University, Vilnius, Lithuania; 98 National Centre for Particle Physics, Universiti Malaya, Kuala Lumpur, Malaysia; 99 Centro de Investigacion y de Estudios Avanzados del IPN, Mexico City, Mexico; 100 Universidad Iberoamericana, Mexico City, Mexico; 101 Benemerita Universidad Autonoma de Puebla, Puebla, Mexico; 102 Universidad Autónoma de San Luis Potosí, San Luis Potosí, Mexico; 103 University of Auckland, Auckland, New Zealand; 104 University of Canterbury, Christchurch, New Zealand; 105 National Centre for Physics, Quaid-I-Azam University, Islamabad, Pakistan; 106 National Centre for Nuclear Research, Swierk, Poland; 107 Institute of Experimental Physics, Faculty of Physics, University of Warsaw, Warsaw, Poland; 108 Laboratório de Instrumentação e Física Experimental de Partículas, Lisboa, Portugal; 109 Joint Institute for Nuclear Research, Dubna, Russia; 110 Petersburg Nuclear Physics Institute, Gatchina, St. Petersburg Russia; 111 Institute for Nuclear Research, Moscow, Russia; 112 Institute for Theoretical and Experimental Physics, Moscow, Russia; 113 P.N. Lebedev Physical Institute, Moscow, Russia; 114 Skobeltsyn Institute of Nuclear Physics, Lomonosov Moscow State University, Moscow, Russia; 115 State Research Center of Russian Federation, Institute for High Energy Physics, Protvino, Russia; 116 Faculty of Physics and Vinca Institute of Nuclear Sciences, University of Belgrade, Belgrade, Serbia; 117 Centro de Investigaciones Energéticas Medioambientales y Tecnológicas (CIEMAT), Madrid, Spain; 118 Universidad Autónoma de Madrid, Madrid, Spain; 119 Universidad de Oviedo, Oviedo, Spain; 120 Instituto de Física de Cantabria (IFCA), CSIC-Universidad de Cantabria, Santander, Spain; 121 CERN, European Organization for Nuclear Research, Geneva, Switzerland; 122 Paul Scherrer Institut, Villigen, Switzerland; 123 Institute for Particle Physics, ETH Zurich, Zurich, Switzerland; 124 Universität Zürich, Zurich, Switzerland; 125 National Central University, Chung-Li, Taiwan; 126 National Taiwan University (NTU), Taipei, Taiwan; 127 Chulalongkorn University, Bangkok, Thailand; 128 Cukurova University, Adana, Turkey; 129 Physics Department, Middle East Technical University, Ankara, Turkey; 130 Bogazici University, Istanbul, Turkey; 131 Istanbul Technical University, Istanbul, Turkey; 132 National Scientific Center, Kharkov Institute of Physics and Technology, Kharkov, Ukraine; 133 University of Bristol, Bristol, United Kingdom; 134 Rutherford Appleton Laboratory, Didcot, United Kingdom; 135 Imperial College, London, United Kingdom; 136 Brunel University, Uxbridge, United Kingdom; 137 Baylor University, Waco, USA; 138 The University of Alabama, Tuscaloosa, USA; 139 Boston University, Boston, USA; 140 Brown University, Providence, USA; 141 University of California, Davis, USA; 142 University of California, Los Angeles, USA; 143 University of California, Riverside, Riverside, USA; 144 University of California, San Diego, La Jolla USA; 145 University of California, Santa Barbara, Santa Barbara, USA; 146 California Institute of Technology, Pasadena, USA; 147 Carnegie Mellon University, Pittsburgh, USA; 148 University of Colorado at Boulder, Boulder, USA; 149 Cornell University, Ithaca, USA; 150 Fairfield University, Fairfield, USA; 151 Fermi National Accelerator Laboratory, Batavia, USA; 152 University of Florida, Gainesville, USA; 153 Florida International University, Miami, USA; 154 Florida State University, Tallahassee, USA; 155 Florida Institute of Technology, Melbourne, USA; 156 University of Illinois at Chicago (UIC), Chicago, USA; 157 The University of Iowa, Iowa City, USA; 158 Johns Hopkins University, Baltimore, USA; 159 The University of Kansas, Lawrence, USA; 160 Kansas State University, Manhattan, USA; 161 Lawrence Livermore National Laboratory, Livermore, USA; 162 University of Maryland, College Park, USA; 163 Massachusetts Institute of Technology, Cambridge, USA; 164 University of Minnesota, Minneapolis, USA; 165 University of Mississippi, Oxford, USA; 166 University of Nebraska-Lincoln, Lincoln, USA; 167 State University of New York at Buffalo, Buffalo, USA; 168 Northeastern University, Boston, USA; 169 Northwestern University, Evanston, USA; 170 University of Notre Dame, Notre Dame, USA; 171 The Ohio State University, Columbus, USA; 172 Princeton University, Princeton, USA; 173 University of Puerto Rico, Mayaguez, USA; 174 Purdue University, West Lafayette, USA; 175 Purdue University Calumet, Hammond, USA; 176 Rice University, Houston, USA; 177 University of Rochester, Rochester, USA; 178 The Rockefeller University, New York, USA; 179 Rutgers, The State University of New Jersey, Piscataway, USA; 180 University of Tennessee, Knoxville, USA; 181 Texas A&M University, College Station, USA; 182 Texas Tech University, Lubbock, USA; 183 Vanderbilt University, Nashville, USA; 184 University of Virginia, Charlottesville, USA; 185 Wayne State University, Detroit, USA; 186 University of Wisconsin, Madison, USA; 187 CERN, Geneva Switzerland

## Abstract

Measurements are reported of the WZ and ZZ production cross sections in proton-proton collisions at $\sqrt{s} = 8$ $\,\text {TeV}$ in final states where one Z boson decays to b-tagged jets. The other gauge boson, either W or Z, is detected through its leptonic decay (either $\mathrm {W}\rightarrow \mathrm {e}\nu$, $\mathrm {\mu }\nu$ or $\mathrm {Z}\rightarrow \mathrm {e}^+\mathrm {e}^-$, $\mathrm {\mu ^+}\mathrm {\mu ^-}$, or ${\nu }\overline{\nu }$). The results are based on data corresponding to an integrated luminosity of 18.9 fb$^{-1}$ collected with the CMS detector at the Large Hadron Collider. The measured cross sections, $\sigma (\mathrm {p}\mathrm {p}\rightarrow \mathrm {W}\mathrm {Z}) = 30.7 \pm 9.3\,\text {(stat.)} \pm 7.1\,\text {(syst.)} \pm 4.1\,\text {(th.)} \pm 1.0\,\text {(lum.)} \,\text {pb}$ and $\sigma (\mathrm {p}\mathrm {p}\rightarrow \mathrm {Z}\mathrm {Z}) = 6.5 \pm 1.7\,\text {(stat.)} \pm 1.0\,\text {(syst.)} \pm 0.9\,\text {(th.)} \pm 0.2\,\text {(lum.)} \,\text {pb}$, are consistent with next-to-leading order quantum chromodynamics calculations.

## Introduction

The study of WZ and ZZ (referred to collectively as VZ) diboson production in proton-proton collisions provides an important test of the gauge sector of the standard model (SM). In $\mathrm {p}$ $\mathrm {p}$ collisions at $\sqrt{s} = 8\,\text {TeV}$, the predicted cross sections are $\sigma (\mathrm {p}\mathrm {p}\rightarrow \mathrm {W}\mathrm {Z})= 22.3 \pm 1.1\,\text {pb}$ and $\sigma (\mathrm {p}\mathrm {p}\rightarrow \mathrm {Z}\mathrm {Z})= 7.7 \pm 0.4\,\text {pb}$ at next-to-leading order (NLO) in quantum chromodynamics (QCD) [1]. A significant deviation from these theoretical values would indicate contributions from physics beyond the SM. Both processes constitute important backgrounds to the associated production of V and standard model Higgs (H) bosons, especially in those channels involving ${\hbox {H}} \rightarrow {\mathrm {b}}\overline{{\mathrm {b}}}$ decays. The production rate of two vector bosons in $\mathrm {p}$ $\mathrm {p}$ collisions at the Large Hadron Collider (LHC) has been measured by the ATLAS and Compact Muon Solenoid (CMS) Collaborations in all-leptonic WZ and ZZ decay modes [2–5].

We present a measurement of the VZ production cross sections in the $\mathrm {V} \mathrm {Z}\rightarrow \mathrm {V} {\mathrm {b}}\overline{{\mathrm {b}}}$ decay mode, where the V decays leptonically: $\mathrm {Z}\rightarrow {\nu }\overline{\nu }$, $\mathrm {W}^{\pm }\rightarrow \ell ^{\pm }{\nu }$, and $\mathrm {Z}\rightarrow \ell ^{+}\ell ^{-}$, with $\ell$ corresponding to either $\mathrm {e}$ or $\mu$. Contributions from $\mathrm {W}\rightarrow \mathrm {\tau }{\nu }$ with leptonic $\tau$ decays are included in the $\mathrm {W}^{\pm }\rightarrow \ell ^{\pm }{\nu }$ channels. The analysis uses final states with no charged leptons (0-lepton), single lepton (1-lepton), or dilepton (2-lepton) events with electron and muon channels analyzed separately. The Z boson decays to ${\mathrm {b}}$ quarks are selected by requiring the presence of two b-tagged jets. The results are based on data corresponding to an integrated luminosity of 18.9 fb$^{-1}$ collected with the CMS detector at the LHC. Two methods are used in the analysis, one involves a fit to the output of a multivariate discriminant, and the other a fit to the two-jet mass ($m_{{\mathrm {b}}\overline{{\mathrm {b}}}}$) distribution. The cross sections are calculated simultaneously for WZ and ZZ production at transverse momenta of the accompanying V of $p_{\mathrm {T}} ^{\mathrm {V}}> 100\,\text {GeV}$, for Z boson masses falling within the window $60<M_{\mathrm {Z}}<120\,\text {GeV}$. The latter requirement assures a uniform treatment of interference with background processes. Approximately 15 % of the WZ and 14 % of the ZZ total inclusive cross sections are contained within their respective regions of acceptance for $p_{\mathrm {T}} ^{\mathrm {V}}> 100\,\text {GeV}$, as calculated using several event generators discussed in the following section. The 1-lepton channel is sensitive almost exclusively to WZ production, while the 2-lepton modes are restricted to the ZZ process. The channel with no charged leptons is sensitive to both production modes, with ZZ and WZ channels contributing 70 % and 30 %, respectively, to these events. The 0-lepton WZ events contribute primarily when the lepton from $\mathrm {W}^{\pm }\rightarrow \ell ^{\pm }{\nu }$ falls outside of acceptance.

## CMS detector, triggering, object reconstruction and event simulation

A description of the CMS detector can be found in Ref. [6]. Particles produced in $\mathrm {p}\mathrm {p}$ collisions are detected in the pseudorapidity range $|\eta |< 5$, where $\eta = -\ln [\tan (\theta /2)]$, and $\theta$ is the polar angle relative to the direction of the counterclockwise circulating proton beam. The CMS detector comprises a superconducting solenoid, providing a uniform axial magnetic field of 3.8$\,\text {T}$ over a cylindrical region that is 12.5$\,\text {m}$ long and 6$\,\text {m}$ in diameter. The magnetic volume contains a silicon pixel and strip tracking system ($|\eta | < 2.5$), surrounded by a lead tungstate crystal electromagnetic calorimeter (ECAL) and a brass/scintillator hadronic calorimeter (HCAL) at $|\eta | < 3.0$. A steel/quartz-fiber Cherenkov calorimeter extends the coverage to $|\eta | = 5$. The steel flux-return yoke outside the solenoid is instrumented with gas-ionization detectors used to identify muons at $|\eta | < 2.4$.

The 1-lepton channels rely on several single-lepton triggers with $p_{\mathrm {T}}$ thresholds between 17 and $30\,\text {GeV}$ and restrictive lepton identification. The 2-lepton channels use the same single-muon triggers for selecting the $\mathrm {Z}\rightarrow \mathrm {\mu ^+}\mathrm {\mu ^-}$ events and 2-electron triggers with $p_{\mathrm {T}}$ thresholds of 17 and 8$\,\text {GeV}$ for the electron of higher and lower $p_{\mathrm {T}}$, respectively, and with more restrictive isolation requirements for selecting the $\mathrm {Z}\rightarrow \mathrm {e}^+\mathrm {e}^-$ events.

A combination of several triggers is used for the events without charged leptons: all triggers require $E_{\mathrm {T}}^{\text {miss}}$ to be above a given threshold, such that the trigger efficiency ranges from 70 to 99 % for $E_{\mathrm {T}}^{\text {miss}} =100\,\text {GeV}$ to $170\,\text {GeV}$, respectively.

Electron reconstruction requires a match of a cluster in the ECAL to a track reconstructed in the silicon tracker [7–9]. Electron identification relies on a multivariate technique that combines observables sensitive to the amount of bremsstrahlung emitted along the electron trajectory, the match in position and energy of the electron trajectory with the associated cluster, as well as the energy distribution in the cluster. Additional requirements are imposed to minimize background from electrons produced through photons converting into $\mathrm {e}^+\mathrm {e}^-$ pair while traversing the tracker material. Electron candidates are considered if observed in the pseudorapidity range $|\eta | < 2.5$ but excluding the transition regions at $1.44 < |\eta |< 1.57$ between the ECAL barrel and endcaps.

Muons are reconstructed using two algorithms [10]: one in which tracks in the silicon tracker are matched to signals in the muon chambers, and another in which a global fit is performed to the track that is seeded by signals detected in the outer muon system. The muon candidates are required to be reconstructed by both algorithms. Additional identification criteria are imposed on muon candidates to reduce the fraction of tracks misidentified as muons. These include the number of hits reconstructed in the tracker and in the muon system, the quality of the global fit to a muon trajectory, and its consistency with originating from the primary vertex. Muon candidates are finally required to fall in the $|\eta | < 2.4$ range.

Jets are reconstructed from particle-flow [11, 12] objects using the anti-$k_{\mathrm {T}}$ jet clustering algorithm [13], with a distance parameter of 0.5, as implemented in the fastjet package [14, 15]. Each jet is required to lie within $|\eta | < 2.5$ and have $p_{\mathrm {T}} > 20\,\text {GeV}$. Jet energy corrections are applied as a function of $\eta$ and $p_{\mathrm {T}}$ of the jet [16]. The imbalance in transverse momentum (often referred to as “missing transverse energy vector”) is calculated as the negative of the vectorial sum of the ${\varvec{p}}_{\mathrm {T}}$ of all particle-flow objects identified in the event, and the magnitude of this vector is referred to as $E_{\mathrm {T}}^{\text {miss}}$. The procedures of Ref. [17] are applied on an event-by-event basis to mitigate the effects of multiple interactions per beam crossing (pileup).

The CMS combined secondary-vertex (CSV) b-tagging algorithm [18] is used to identify jets that are likely to originate from the hadronization of b quarks. This algorithm combines the information about track impact parameters and secondary vertices in a discriminant that distinguishes ${\mathrm {b}}$ jets from jets originating from light quarks, gluons, or $\mathrm {c}$ quarks. The output of the CSV algorithm is a continuous discriminator with a value in the range 0 to 1, where typical thresholds for ${\mathrm {b}}$ jet selection range from loose ($\approx$0.2) to tight ($\approx$0.9). Depending on the chosen CSV threshold, the efficiencies for tagging jets originating from ${\mathrm {b}}$ quarks range from 50 % (tight) to 75 % (loose), while the misidentification rates for $\mathrm {c}$ quarks range from 5 % (tight) to 25 % (loose) and for light quarks or gluons range from 0.2 % (tight) to 3 % (loose).

The b-jet energy resolution is improved by applying multivariate regression techniques similar to those used in the CDF experiment [19]. An additional correction, beyond the standard CMS jet energy corrections, is derived from simulated events to recalibrate each b-tagged jet with the generated ${\mathrm {b}}$ quark energy. This involves a specialized boosted decision tree (BDT) [20, 21] trained on simulated signal events, with inputs that include information on jet structure, such as information about individual tracks, jet constituents, information on semileptonic b-hadron decays, and the presence of any low-$p_{\mathrm {T}}$ leptons. The BDT correction, identical to that used in Ref. [17], improves the resolution on the mass of the ${\mathrm {b}}\overline{{\mathrm {b}}}$ system by ${\approx }$15 %, resulting in an increase in the sensitivity of the analysis of 10–20 %, depending on the specific channel. The $\mathrm {Z}\rightarrow {\mathrm {b}}\overline{{\mathrm {b}}}$ invariant mass resolution after this correction is ${\approx }$10 %.

Simulated samples of events are produced using several event generators, and the response of the CMS detector is modeled using the Geant4 program [22]. The MadGraph 5.1 [23] generator is used to generate the diboson signals, as well as the background from W+jets, Z+jets, and $\mathrm {t}\overline{\mathrm {t}}$ events. The single-top-quark samples are generated with powheg  [24–27], and generic multijet samples using pythia 6.4 [28]. VH event samples with a SM H boson mass of $m_{{\hbox {H}}}= 125\,\text {GeV}$ are also produced using the powheg  [29] event generator interfaced to herwig ++ [30] for parton showering and hadronization. The NLO MSTW2008 set [31] of parton distribution functions (PDF) is used to produce the NLO powheg samples, while the leading-order (LO) CTEQ6L1 set [32] is used for the events that correspond to LO calculations. The Z2Star tune [33] is used to parametrize the underlying event. Corrections to account for differences in efficiencies between data and simulation are measured using data using a tag and probe technique [34], and applied as individual weights to each of the simulated events.

## Event selection

We use the analysis techniques developed in the CMS VH studies of Ref. [17]. Event selection is based on the reconstruction of a vector boson that decays leptonically in association with the Z boson that decays into two b-tagged jets. Dominant backgrounds to VZ production include V+${\mathrm {b}}$ jets, V+light flavor (LF = $\mathrm {u}$ $\mathrm {d}$ $\mathrm {s}$ $\mathrm {c}$ quark or gluon) jets, $\mathrm {t}\overline{\mathrm {t}}$, single-top-quark, generic multijet, and H boson production. In general, b-tagging reduces the contributions from LF events, and counting additional jet activity is used to reduce background from $\mathrm {t}\overline{\mathrm {t}}$ and single-top-quark events. Finally, the value of $m_{{\mathrm {b}}\overline{{\mathrm {b}}}}$ provides a way to distinguish VZ from V+${\mathrm {b}}$ and SM VH production, as discussed below.

The reconstruction of a $\mathrm {Z}\rightarrow {\mathrm {b}}\overline{{\mathrm {b}}}$ decay proceeds by selecting two central jets from the primary vertex with $|\eta |<2.5$, each with a $p_{\mathrm {T}}$ above some chosen threshold, and defining the ${\mathrm {b}}\overline{{\mathrm {b}}}$ candidate as the jet pair with largest vectorial sum of transverse momenta (${p_{\mathrm {T}}}^{{\mathrm {b}}\overline{{\mathrm {b}}}}$). This combination is very efficient for $p_{\mathrm {T}} ^{\mathrm {V}}>100\,\text {GeV}$ without biasing the differential distribution of the background, and also defines the two-jet mass $m_{{\mathrm {b}}\overline{{\mathrm {b}}}}$, which is required to be $<250\,\text {GeV}$. The two selected jets are also required to be tagged as ${\mathrm {b}}$ jets, with a value of the CSV discriminator that depends on the specific nature of the event.

Candidate $\mathrm {W}^{\pm }\rightarrow \ell ^{\pm }{\nu }$ decays in WZ events are identified through the presence of a single isolated lepton and significant $E_{\mathrm {T}}^{\text {miss}}$. Electrons and muons are required to have $p_{\mathrm {T}} >30\,\text {GeV}$ and $p_{\mathrm {T}} >20\,\text {GeV}$, respectively. To reduce contamination from generic multijet processes, the $E_{\mathrm {T}}^{\text {miss}}$ is required to be $>45\,\text {GeV}$. In addition, the azimuthal angle ($\phi$) between the $E_{\mathrm {T}}^{\text {miss}}$ vector and the lepton is required to be $<\pi /2$. At least two jets with $p_{\mathrm {T}} > 30\,\text {GeV}$ and a moderate CSV discriminator value are required to define the $\mathrm {Z}\rightarrow {\mathrm {b}}\overline{{\mathrm {b}}}$ candidate.

Candidate $\mathrm {Z}\rightarrow \ell ^{+}\ell ^{-}$ decays in ZZ events are reconstructed by combining isolated, oppositely charged pairs of electrons or muons, with a dilepton invariant mass of $75<m_{\ell \ell }<105\,\text {GeV}$. The $p_{\mathrm {T}}$ of each lepton is required to be $>20\,\text {GeV}$. The two jets of the $\mathrm {Z}\rightarrow {\mathrm {b}}\overline{{\mathrm {b}}}$ candidate must pass a loose CSV discriminator value, which is optimized in simulated events for increasing the sensitivity of the analysis.

The identification of $\mathrm {Z}\rightarrow {\nu }\overline{\nu }$ decays in ZZ events requires $E_{\mathrm {T}}^{\text {miss}} >100\,\text {GeV}$ in the event, and at least one of the ${\mathrm {b}}$ jets with $p_{\mathrm {T}} > 60\,\text {GeV}$ and the other with $p_{\mathrm {T}} > 30\,\text {GeV}$ to form a $\mathrm {Z}\rightarrow {\mathrm {b}}\overline{{\mathrm {b}}}$ candidate. Moderate CSV requirements are applied on both jets. Two additional event requirements are imposed to reduce the multijet background in which $E_{\mathrm {T}}^{\text {miss}}$ originates from mismeasured jet energies. First, a $\Delta \phi (E_{\mathrm {T}}^{\text {miss}},\text {jet})$ $>0.5$ radians requirement is applied on the azimuthal angle between the direction of $E_{\mathrm {T}}^{\text {miss}}$ and the $p_{\mathrm {T}}$ of the jet closest in $\phi$ that satisfies $|\eta |<2.5$ and $p_{\mathrm {T}} >25\,\text {GeV}$. The second requirement is that the azimuthal angle between the direction of ${E_{\mathrm {T}}^{\text {miss}}}^\text {(trks)}$, as calculated from only the charged tracks that satisfy $p_{\mathrm {T}} >0.5\,\text {GeV}$ and $| \eta |<2.5$, and the direction of the full $E_{\mathrm {T}}^{\text {miss}}$ has $\Delta \phi (E_{\mathrm {T}}^{\text {miss}},{E_{\mathrm {T}}^{\text {miss}}}^{\text{( }trks)})<0.5$ radians. Finally, to reduce background from $\mathrm {t}\overline{\mathrm {t}}$ events in the 1-lepton and 0-lepton channels, events that contain any additional isolated leptons with $p_{\mathrm {T}} >20\,\text {GeV}$ are rejected.

### Multivariate analysis

The signal region is defined by events that satisfy the V and Z boson reconstruction criteria described above. To optimize the significance of the signal as well as the ${\mathrm {b}}\overline{{\mathrm {b}}}$ mass resolution, events are classified into different regions of the V boson transverse momentum. In particular, we define three regions for the 1-lepton channels: (i) $100<p_{\mathrm {T}} ^{\mathrm {V}}<130\,\text {GeV}$, (ii)  $130<p_{\mathrm {T}} ^{\mathrm {V}}<180\,\text {GeV}$, and (iii)  $p_{\mathrm {T}} ^{\mathrm {V}}>180\,\text {GeV}$. A single inclusive region of $p_{\mathrm {T}} ^{\mathrm {V}}>100\,\text {GeV}$ is defined for the 2-lepton channels. Three regions for the channel without charged leptons are defined by (i) $100<p_{\mathrm {T}} ^{\mathrm {V}}<130\,\text {GeV}$, (ii) $130<p_{\mathrm {T}} ^{\mathrm {V}}<170\,\text {GeV}$, and (iii) $p_{\mathrm {T}} ^{\mathrm {V}}>170\,\text {GeV}$. For regions (i) and (ii), the requirement on $\Delta \phi (E_{\mathrm {T}}^{\text {miss}},\text {jet})$ is tightened to $\Delta \phi (E_{\mathrm {T}}^{\text {miss}},\text {jet})>0.7$ radians. To reduce background in the region of smallest $p_{\mathrm {T}} ^{\mathrm {V}}$, the $E_{\mathrm {T}}^{\text {miss}}$ significance (defined as the ratio of $E_{\mathrm {T}}^{\text {miss}}$ to the square root of the total transverse energy deposited in the calorimeter) is required to be ${>}3 \sqrt{\text {GeV}}$.

To better discriminate between signals and background, the final stage of the analysis introduces a BDT discriminant trained on simulated samples for signal and all background processes. The set of input variables is identical to the one used in Ref. [17], and includes the mass of the ${\mathrm {b}}\overline{{\mathrm {b}}}$ system, the number of additional jets beyond the ${\mathrm {b}}$ and $\overline{{\mathrm {b}}}$ candidates ($N_{\mathrm {aj}}$), the value of CSV for the ${\mathrm {b}}\overline{{\mathrm {b}}}$ jets with $\mathrm {CSV}_{\text {min}}$ specifying the smaller value and $\mathrm {CSV}_{\text {max}}$ the larger one, and the distance in $\eta$-$\phi$ between the ${\mathrm {b}}$ and $\overline{{\mathrm {b}}}$ jet axes, $\Delta R({\mathrm {b}}\overline{{\mathrm {b}}})= \sqrt{{\left( \Delta \phi \right) ^2+\left( \Delta \eta \right) ^2}}$.

**Fig. 1:**
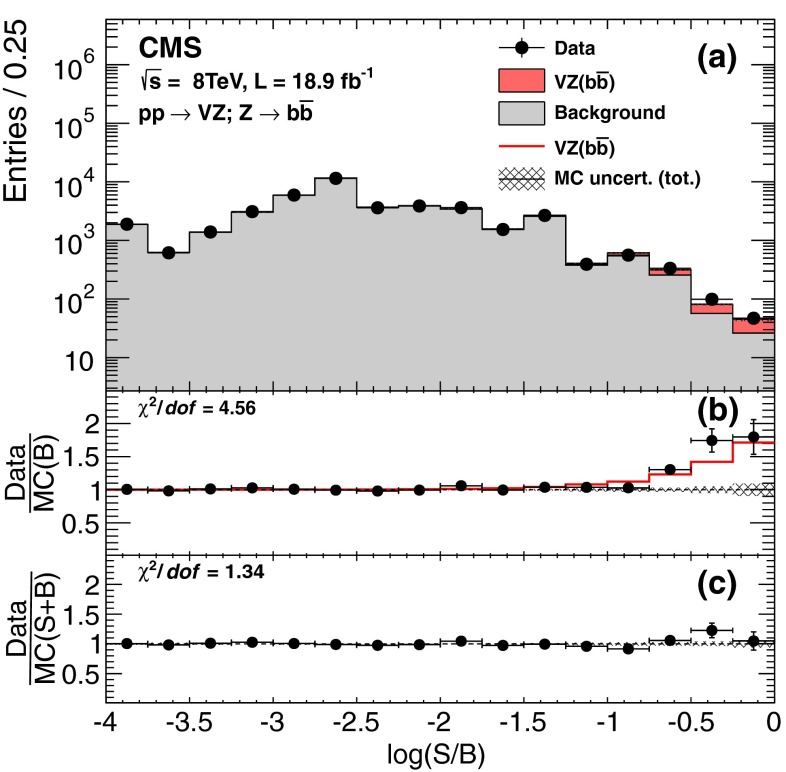
**(a)** Combined distribution for all channels in the value of the logarithm of the ratio of signal to background (S/B) discriminants in data and in Monte Carlo (MC) simulations, based on the outputs of the S and B BDT discriminants for each event. The two bottom panels display **(b)** the ratio of the data and of the SM expectation relative to the background-only hypothesis, and **(c)** data relative to the expected sum of background and VZ signal. The error bars and the cross-hatched regions reflect total uncertainties at 68 % confidence level

Figure 1(a) displays the combined differential distribution for events from all channels as a function of the logarithm of the signal-to-background (S/B) ratio of the values of the output of the corresponding S and B contributions to the BDT discriminants of each event. Panel (b) gives the ratio of the data (black points) to the SM expectation (histogram) relative to the background-only hypothesis, while panel (c) gives the ratio to the expectation from the SM, including the VZ contribution. The excess observed in bins with largest S/B is clearly consistent with what is expected for VZ production in the SM.

### Two-jet mass analysis

As a cross-check of the multivariate analysis, we perform a simpler analysis based on the $m_{{\mathrm {b}}\overline{{\mathrm {b}}}}$ distribution of the reconstructed ${\mathrm {b}}\overline{{\mathrm {b}}}$ jets of the hypothesized Z boson. The signal region is defined by events that satisfy the V and Z boson reconstruction criteria used in the multivariate analysis. Events are again classified according to $p_{\mathrm {T}} ^{\mathrm {V}}$, and, in addition, more restrictive selections are introduced than in the multivariate analysis, because the single variable $m_{{\mathrm {b}}\overline{{\mathrm {b}}}}$ is not a sufficiently sensitive discriminant.

In the 0-lepton and 1-lepton channels, the b-tagging requirements are tightened, respectively, to a tight $\mathrm {CSV}_{\text {max}}$ and a medium $\mathrm {CSV}_{\text {min}}$. A veto is also imposed on any additional jets, and $\Delta \phi (\mathrm {V},\mathrm {Z})$ is required to be ${>}2.95$ radians. The regions of $100<p_{\mathrm {T}} ^{\mathrm {V}}<130\,\text {GeV}$, $130<p_{\mathrm {T}} ^{\mathrm {V}}<180\,\text {GeV}$, and $p_{\mathrm {T}} ^{\mathrm {V}}>180\,\text {GeV}$ are used to analyze the 1-muon channel, and the regions for the 1-electron channel are defined as $100<p_{\mathrm {T}} ^{\mathrm {V}}<150\,\text {GeV}$ and $p_{\mathrm {T}} ^{\mathrm {V}}>150\,\text {GeV}$. The selected regions for the 0-lepton channel are identical in $p_{\mathrm {T}} ^{\mathrm {V}}$ to the requirements used in the multivariate analysis, but we define ranges of ${p_{\mathrm {T}}}^{{\mathrm {b}}\overline{{\mathrm {b}}}}>110\,\text {GeV}$, ${p_{\mathrm {T}}}^{{\mathrm {b}}\overline{{\mathrm {b}}}}>140\,\text {GeV}$, and ${p_{\mathrm {T}}}^{{\mathrm {b}}\overline{{\mathrm {b}}}}>190\,\text {GeV}$, and impose an additional threshold for the jet of highest $p_{\mathrm {T}}$ of ${>}80\,\text {GeV}$ for the region of ${p_{\mathrm {T}}}^{{\mathrm {b}}\overline{{\mathrm {b}}}}>140\,\text {GeV}$. For the 2-lepton channels, the $p_{\mathrm {T}} ^{\mathrm {V}}$ ranges are defined by $100<p_{\mathrm {T}} ^{\mathrm {V}}<150\,\text {GeV}$ and $p_{\mathrm {T}} ^{\mathrm {V}}>150\,\text {GeV}$, and, in addition, we require medium $\mathrm {CSV}_{\text {max}}$ and moderate $\mathrm {CSV}_{\text {min}}$ thresholds, and $E_{\mathrm {T}}^{\text {miss}} < 60\,\text {GeV}$.

**Fig. 2:**
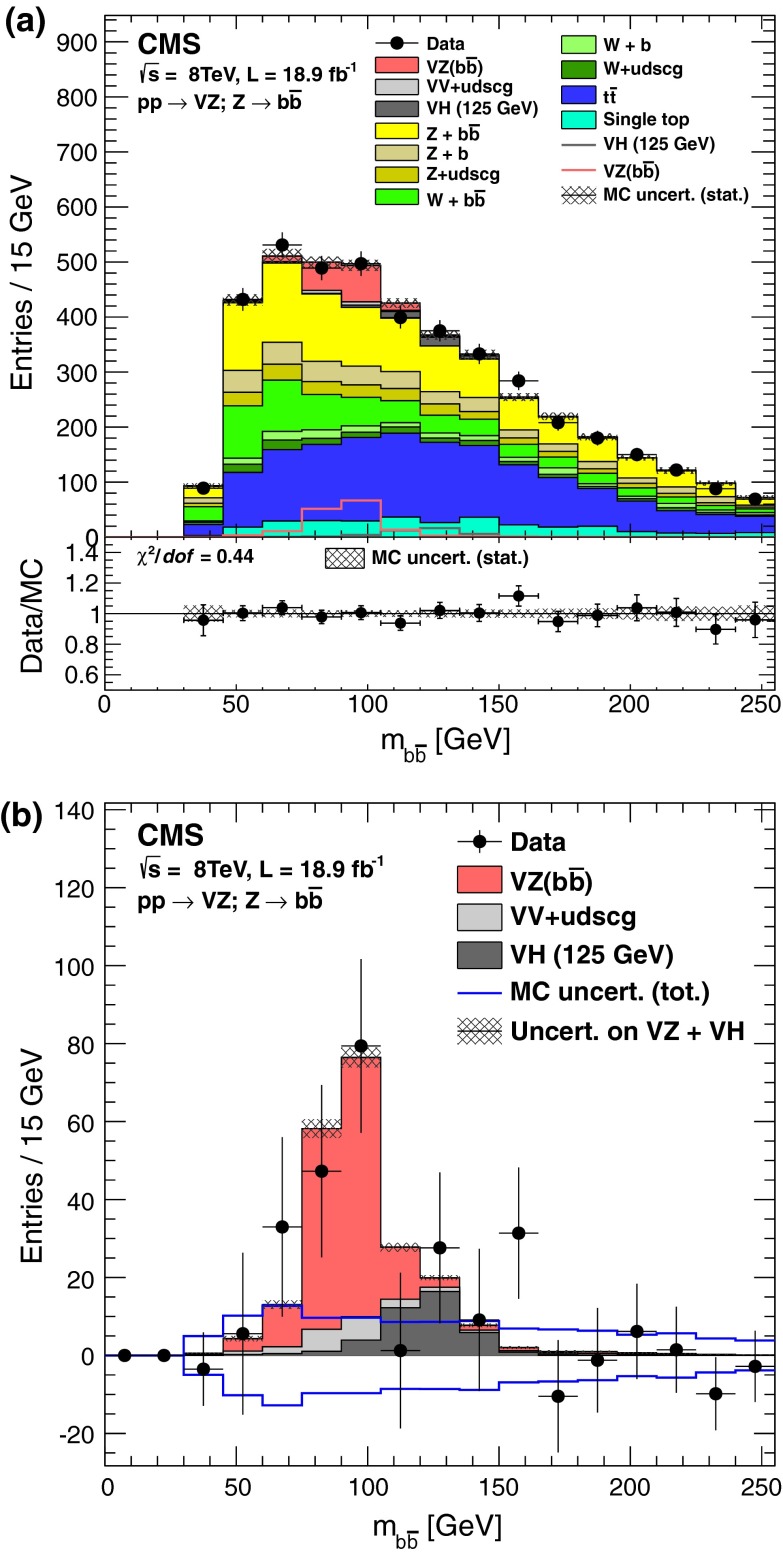
**(a)** The combined ${\mathrm {b}}\overline{{\mathrm {b}}}$ invariant mass distribution for all channels, compared to MC simulation of SM contributions. **(b)** Same distribution as in (a), but with all backgrounds to VZ production, except for the VH contribution, subtracted. The contributions from backgrounds and signal are summed cumulatively. The expectations for the sum of VZ signal and background from VH production are also shown superimposed. The error bars and cross-hatched regions reflect statistical uncertainties at 68 % confidence level

Figure 2(a) combines events from all channels into a single $m_{{\mathrm {b}}\overline{{\mathrm {b}}}}$ distribution, which is compared to expectations from the SM. Figure 2(b) shows the same distribution, but after subtracting all SM contributions except for the VZ signals and VH backgrounds. The VZ signal is clearly visible, with a yield compatible to that expected in the SM.

## Background calibration regions and systematic uncertainties

Calibration regions in data are used to validate the simulated distributions used to build the BDT discriminants, as well as to correct normalizations of the major background contributions from W and Z bosons produced in association with jets (LF or ${\mathrm {b}}$ quarks) and $\mathrm {t}\overline{\mathrm {t}}$ production. These calibration regions are identical to those of Ref. [17], and typically involve inversion of b-tag selection criteria and two-jet mass sidebands around the signal region. A set of simultaneous fits is then performed to distributions of discriminating variables in the calibration regions, separately for each channel, to obtain consistent scale factors that are used to adjust the yields from simulated events. These scale factors account not only for discrepancies between predicted cross sections and data, but also for any residual differences in the selection of physical objects. Separate scale factors are consequently applied for each of the background processes in the different channels. For the backgrounds from V+jets, the calibration regions are enriched in either ${\mathrm {b}}$ or LF jets. Uncertainties in the scale factors include statistical components arising from the fits to the discriminant (affected by the finite size of the data and MC samples), and systematic uncertainties originating from ${\mathrm {b}}$ tagging, jet energy scale, and jet energy resolution. The numerical values of the scale factors are close to unity and their uncertainties (3–50 %) are identical to those of Ref. [17].

The systematic uncertainties considered in the measurement of the cross section using the multivariate analysis are summarized in Table 1. The two columns give the uncertainty in the “signal strength” $\mu$ for the WZ and ZZ processes, which corresponds to the ratio of the observed yield relative to the yield expected from the SM. Each systematic uncertainty is represented by a nuisance parameter and profiled in the combined fit. To evaluate the impact of individual uncertainties a fit to a simulated pseudo-dataset is performed removing individual nuisance parameters.

**Table 1:** Sources of systematic uncertainty, including whether they affect the distribution (dist) or normalization (norm) of the BDT output, and their relative contributions to the expected uncertainty in the signal strengths $\mu _{\mathrm {W}\mathrm {Z}}$ and $\mu _{\mathrm {Z}\mathrm {Z}}$ after fitting the model

Source of uncertainty	Type	Individual contributions to uncertainty	
		$\mu _{\mathrm {W}\mathrm {Z}}$ (%)	$\mu _{\mathrm {Z}\mathrm {Z}}$ (%)
Luminosity	norm	3.3	3.2
Lepton efficiency and trigger	norm	1.9	0.6
0-lepton triggers	dist	$-$	1.6
Jet energy scale	dist	7.2	6.4
Jet energy resolution	dist	6.1	5.9
$E_{\mathrm {T}}^{\text {miss}}$	dist	3.3	1.8
b tagging	dist	7.7	5.7
VZ cross section (theory)	norm	13.4	13.4
Monte Carlo statistics	dist	5.5	3.6
Backgrounds (from data)	norm	12.5	11.5
Single-top and VH (from simulation)	norm	1.9	$-$
MC modeling of V+jets and $\mathrm {t}\overline{\mathrm {t}}$	dist	4.7	4.8

Theoretical uncertainties in the acceptances are evaluated using the mcfm [1] generator by changing the QCD factorization and renormalization scales up and down by a factor of two relative to the default scales of $\mu _R = \mu _F = m_Z$. The impact of uncertainties in PDF and $\alpha _s$ on the cross section and acceptance of the VZ signal are evaluated following the PDF4LHC prescription [35, 36], using CT10 [37], MSTW08 [31], and NNPDF2.0 [38] sets of PDF, and the combined uncertainty is found to be 5 % for both WZ and ZZ production. Because of the large $p_{\mathrm {T}} ^{\mathrm {V}}$ values required in this analysis, the results are sensitive to electroweak (EW) and NNLO QCD corrections, both of which can be significant. Since the exact corrections for the VZ process are not available, we use the NLO EW [39–41] and next-to-next-to-leading-order (NNLO) QCD [42] corrections to VH production, and apply these to the VZ channel, because they are expected to be similar for the two processes. Based on the size of the correction, an additional 10 % uncertainty is assigned to the inclusive cross section to account for the extrapolation to the $p_{\mathrm {T}} ^{\mathrm {V}}<100\,\text {GeV}$ region.

The uncertainty in CMS luminosity is estimated to be 2.6 % [43]. Muon and electron triggering, reconstruction, and identification efficiencies are determined in data from samples of $\mathrm {Z}\rightarrow \ell ^{+}\ell ^{-}$ decays. The uncertainty in the lepton yields due to trigger inefficiency is 2 % per lepton, as is the uncertainty in lepton identification efficiency. The parameters describing the turn-on in the trigger efficiency in the 0-lepton channel are varied within their statistical uncertainties for different assumptions on the methods used to derive the efficiency. The estimated uncertainty is 3 %.

The jet energy scale is also varied within its uncertainty as a function of jet $p_{\mathrm {T}}$ and $\eta$, and the efficiency of the selections is then recomputed to assess the dependence on these variables. The effect of this uncertainty on the jet energy resolution is evaluated by smearing the jet energies according to their measured uncertainties, a process that affects both the normalization and distribution of events. An uncertainty of 3 % is assigned to the yields of all processes in the 0-lepton and 1-lepton channels due to uncertainties related to $E_{\mathrm {T}}^{\text {miss}}$, such as its scale and resolution.

Scaling factors to normalize b-tagging in simulation to that in data (measured in ${\mathrm {b}}$ enhanced samples of jets that contain muons) are applied consistently to jets in simulated signal and background events. The measured uncertainties in b-tagging scale factors are 3 % per b-quark jet, 6 % per c-quark jet, and 15 % per mistagged jet (originating from a gluon or from a light quark) [18]. These translate into uncertainties in yields of 3–15 %, depending on channel and specific process. The BDT output is also affected by the distributions of the CSV output, and an uncertainty is therefore assigned according to ${\pm }1$ standard deviation (SD) variation in yield and shape of the CSV distributions.

Finally, the sizes of the simulated samples, as well as uncertainties in generator-level modeling of V+jets and $\mathrm {t}\overline{\mathrm {t}}$ backgrounds, are taken into account to determine the total uncertainty in the signal strength $\mu$.

## Results

The total cross sections are determined from a simultaneous fit to all final states, constrained by the number of events observed in each category. The likelihood is written as a combination of individual channel likelihoods for the signal and background hypotheses. We extract the best-fit values of the signal strength assuming the SM expectation for the ratio of $\sigma {(\mathrm {W}\mathrm {Z})}/\sigma {(\mathrm {Z}\mathrm {Z})}$ at NLO. Using the baseline multivariate analysis, the VZ process is observed with a statistical significance of 6.3 SD (5.9 SD expected). The measurement corresponds to a signal strength relative to the SM of $\mu = 1.09 {}_{-0.21}^{+0.24}$. The cross-check analysis based on $m_{{\mathrm {b}}\overline{{\mathrm {b}}}}$ yields a significance of 4.1 SD (4.6 SD expected), which corresponds to $\mu = 0.97 {}_{-0.29}^{+0.32}$. In the following, the interpretation refers to the more sensitive multivariate analysis.

**Fig. 3:**
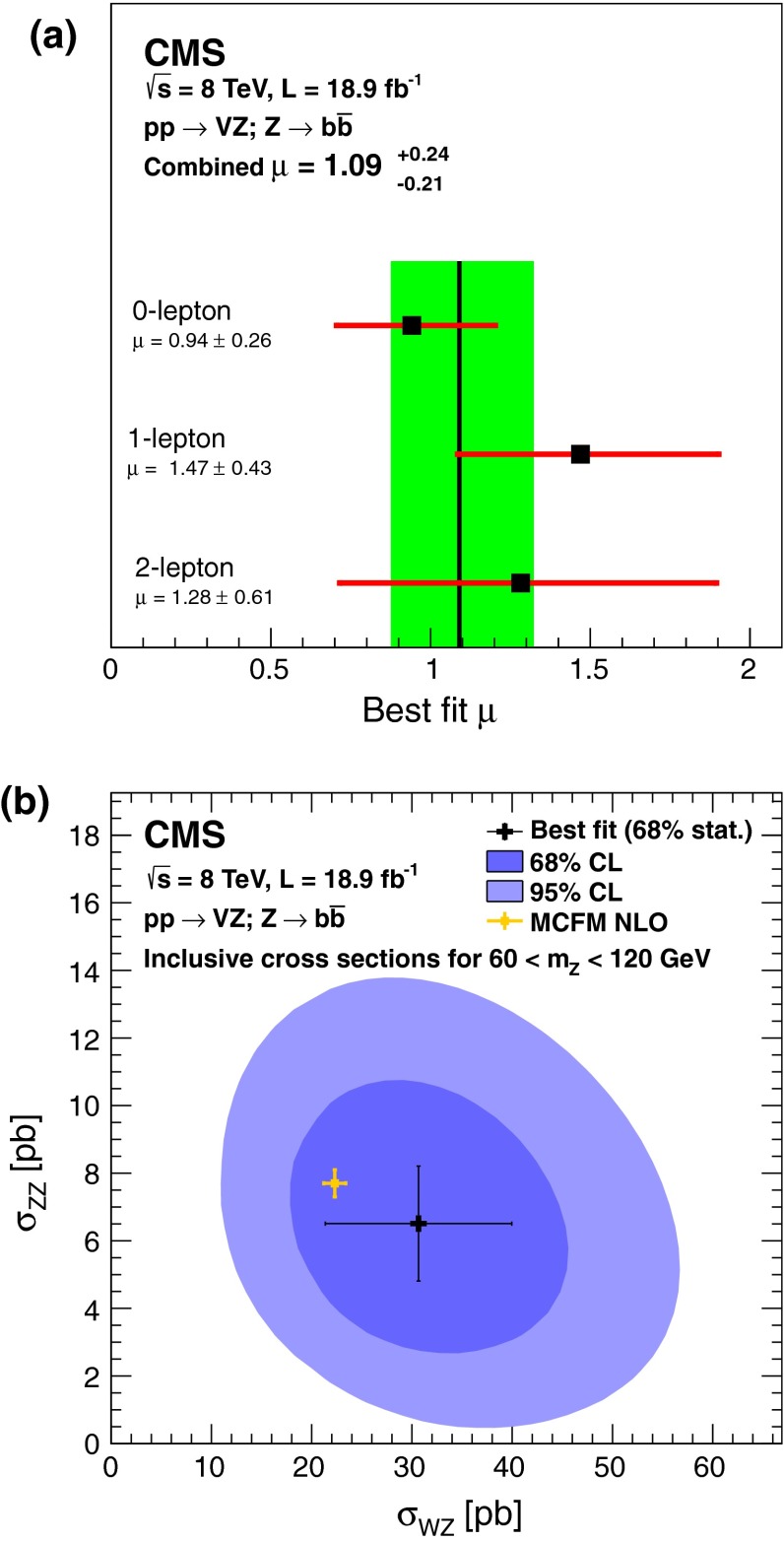
**(a)** Best-fit values of the ratios of the VZ production cross sections, relative to SM predictions for individual channels, and for all channels combined (hatched band). **(b)** Contours of 68 and 95 % confidence level for WZ and ZZ production cross sections. The large cross indicates the best-fit value including its 68 % statistical uncertainty, and the light small cross shows the result for the MCFM NLO calculation

The cross sections extracted from the individual channels are consistent with each other and with the SM predictions, as can be seen in Fig. 3(a). To extract the WZ and ZZ cross sections, a simultaneous fit is performed floating independently the WZ and ZZ contributions, with results displayed in Fig. 3(b). The most likely values are $\mu _{\mathrm {W}\mathrm {Z}} = 1.37 {}_{-0.37}^{+0.42}$ and $\mu _{\mathrm {Z}\mathrm {Z}} = 0.85 {}_{-0.31}^{+0.34}$.

The values for the signal strength are extrapolated to the mass window $60<M_{\mathrm {Z}}<120\,\text {GeV}$ for both the ${\mathrm {b}}\overline{{\mathrm {b}}}$ and lepton pair invariant masses. The resulting cross section for inclusive WZ production is $\sigma (\mathrm {p}\mathrm {p}\rightarrow \mathrm {W}\mathrm {Z}) = 30.7 \pm 9.3\,\text {(stat.)} \pm 7.1\,\text {(syst.)} \pm 4.1\,\text {(th.)} \pm 1.0\,\text {(lum.)} \,\text {pb}$, compared to the theoretical value of $\sigma (\mathrm {p}\mathrm {p}\rightarrow \mathrm {W}\mathrm {Z})= 22.3 \pm 1.1\,\text {pb}$, calculated with mcfm using the MSTW2008 PDF. The ZZ cross section is $\sigma (\mathrm {p}\mathrm {p}\rightarrow \mathrm {Z}\mathrm {Z}) = 6.5 \pm 1.7\,\text {(stat.)} \pm 1.0\,\text {(syst.)} \pm 0.9\,\text {(th.)} \pm 0.2\,\text {(lum.)} \,\text {pb}$, for the same Z-mass window, which can be compared to the theoretical value of $\sigma (\mathrm {p}\mathrm {p}\rightarrow \mathrm {Z}\mathrm {Z})= 7.7 \pm 0.4\,\text {pb}$, also calculated with mcfm using the MSTW2008 PDF. The uncertainties in both theoretical values include uncertainties in the PDF and $\alpha _s$, and those originating from the uncertainty in renormalization and factorization scales. The ZZ cross section is in agreement with CMS measurements using all-leptonic V decays of Ref. [5], which is more precise than this analysis.

The cross sections for $p_{\mathrm {T}} ^{\mathrm {V}}> 100\,\text {GeV}$ and for Z bosons produced in the mass region $60<M_{\mathrm {Z}}<120\,\text {GeV}$ are determined to be $\sigma (\mathrm {p}\mathrm {p}\rightarrow \mathrm {W}\mathrm {Z}) = 4.8 \pm 1.4\,\text {(stat.)} \pm 1.1\,\text {(syst.)} \,\text {pb}$ and $\sigma (\mathrm {p}\mathrm {p}\rightarrow \mathrm {Z}\mathrm {Z}) = 0.90 \pm 0.23\,\text {(stat.)} \pm 0.16\,\text {(syst.)} \,\text {pb}$. The acceptance for this $p_{\mathrm {T}}$ region has smaller theoretical uncertainty, estimated as 1 % using MC signal simulation; the measurements are found in agreement with the NLO mcfm calculations yielding $\sigma (\mathrm {p}\mathrm {p}\rightarrow \mathrm {W}\mathrm {Z})= 3.39 \pm 0.17\,\text {pb}$ and $\sigma (\mathrm {p}\mathrm {p}\rightarrow \mathrm {Z}\mathrm {Z})= 1.03 \pm 0.05\,\text {pb}$.

## Summary

We presented measurements of the inclusive $\mathrm {p}\mathrm {p}\rightarrow \mathrm {V} \mathrm {Z}$ (where V denotes W or Z) cross sections in data recorded by the CMS experiment at the LHC at $\sqrt{s} =8\,\text {TeV}$, corresponding to an integrated luminosity of 18.9 fb$^{-1}$. The measurements are based on $\mathrm {V} \mathrm {Z}\rightarrow \mathrm {V} {\mathrm {b}}\overline{{\mathrm {b}}}$ final states. The decay modes $\mathrm {Z}\rightarrow {\nu }\overline{\nu }$, $\mathrm {W}^{\pm }\rightarrow \ell ^{\pm }{\nu }$, and $\mathrm {Z}\rightarrow \ell ^{+}\ell ^{-}$ ($\ell = \mathrm {e}, \mathrm {\mu }$) are used to identify the accompanying V. We observe $\mathrm {V} \mathrm {Z}\rightarrow \mathrm {V} {\mathrm {b}}\overline{{\mathrm {b}}}$ production with a combined significance of 6.3 standard deviations. The total cross sections, defined for $60<M_{\mathrm {Z}}<120\,\text {GeV}$, are found to be $\sigma (\mathrm {p}\mathrm {p}\rightarrow \mathrm {W}\mathrm {Z}) = 30.7 \pm 9.3\,\text {(stat.)} \pm 7.1\,\text {(syst.)} \pm 4.1\,\text {(th.)} \pm 1.0\,\text {(lum.)} \,\text {pb}$ and $\sigma (\mathrm {p}\mathrm {p}\rightarrow \mathrm {Z}\mathrm {Z}) = 6.5 \pm 1.7\,\text {(stat.)} \pm 1.0\,\text {(syst.)} \pm 0.9\,\text {(th.)} \pm 0.2\,\text {(lum.)} \,\text {pb}$. These values are consistent with the predictions $\sigma (\mathrm {p}\mathrm {p}\rightarrow \mathrm {W}\mathrm {Z})= 22.3 \pm 1.1\,\text {pb}$ and $\sigma (\mathrm {p}\mathrm {p}\rightarrow \mathrm {Z}\mathrm {Z})= 7.7 \pm 0.4\,\text {pb}$ of the standard model at next-to-leading order.
